# Breakthrough whole body energy-specific and tissue-specific photoneutron dosimetry by novel miniature neutron dosimeter/spectrometer

**DOI:** 10.1038/s41598-021-99612-2

**Published:** 2021-10-15

**Authors:** Mehdi Sohrabi, Morteza Ebrahimzadeh Torkamani

**Affiliations:** Health Physics and Dosimetry Research Laboratory, Department of Energy Engineering and Physics, Amirkabir University of Technology, Tehran, Iran

**Keywords:** Physics, Particle physics

## Abstract

Breakthrough whole body energy-specific photoneutron (PN) dosimetry was made in/out-of-field in polyethylene phantom organ surface/depths remote from isocenter of 10 × 10 cm^2^ field prostate cancer therapy in 18 MV X-rays Varian Clinac 2100C medical linear accelerator for PN tissue-specific second primary cancer (PN-SPC) risk estimation. A novel miniature neutron dosimeter/spectrometer with polycarbonate/^10^B/cadmium inserts was invented and applied. Each dosimeter determines seven tissue-specific dose equivalent (mSv)/Gy X-ray dose at each measurement point providing seven major energy-specific responses for beam thermal, albedo thermal, total thermal, total epithermal, total fast, sum of totals (thermal + epithermal) and sum of totals (thermal + epithermal + fast) PNs dose equivalents. The neutron dosimeter is simple, efficient, and unique with high spatial resolution and provides matrix of energy-specific PN dose equivalent (mSv)/Gy X-ray dose on surface and organ depths for tissue-specific PN-SPC risk estimation. The dosimeter also performs like a “miniature neutron spectrometer” and is unique for other applications in health physics in particular individual neutron dosimetry, medical physics, space flights, science and technology.

## Introduction

Medical linear accelerators produce high-energy electrons and X-rays and have found widespread applications worldwide for patient cancer radiotherapy. High-energy X-rays produce undesirable photoneutrons (PNs) when interact with the accelerator head structure materials such as linac target, flattening filter, jaws, multileaf collimator (MLC), and surrounding material. The amount of PNs generated largely depends on the field size and the opening of the MLC for which PN dosimetry has been of major concern^1–7^. In fact, high-energy X-rays with energies ≥ 6 MV generate PNs when interact with the accelerator head structure materials especially beryllium exit window in multi-mode medical accelerators^8^. A recent novel multi-directional neutron spectrometry of 18 MV X-ray beams in a radiotherapy bunker well demonstrated two PN peaks; one thermal PN peak with an epithermal and intermediate energy tail followed by another peak for fast PNs ~1 MeV^9^. Thus, for patients undergoing high energy X-ray therapy, unwanted PN exposures can cause second primary cancer (PN-SPC) risks in different tissues of human body organs in and out of the field even in organs remote from the main beam^10–12^. A review of in and out-of-field X-rays and neutron dose measurements by solid state dosimeters and ion chambers usually in water tanks has focused on importance of estimating SPC risks from PNs and X-rays^11^.

Neutrons are high linear energy transfer (LET) particles which produce high-LET secondary charged particles in particular protons when interact with atomic nuclei in human body. Neutrons have therefore high relative biological effectiveness (RBE) and in turn high radiation weighting factors (W_R_) with a continuous function versus neutron energy which is of high concern in radiation protection of workers and patients; e.g. W_R_ value of 5 for thermal and 20 for 1 MeV neutrons are usually applied^10^. On the other hand, human body tissues have different sensitivities to radiation and thus have different tissue weighting factors (W_T_); e.g. skin, thyroid, gonads, and breast have W_T_ values of 0.01, 0.04, 0.08 and 0.12 respectively^10^.

The rationale for assigning correct radiation W_T_ and W_R_ values in radiation protection as well as for estimating accurately tissue-specific PN-SPC risks of patients undergoing high energy X-ray therapy is to have a neutron dosimeter with some specific neutron dosimetry characteristics. Some characteristics include high energy-specific sensitivity to fast, epithermal and thermal PNs from the beam and also albedo PNs; high spatial resolution for in-depth tissue-specific dose equivalent determination; insensitivity to high doses of low-LET radiation (X, γ, β) and non-ionizing radiation; little/negligible post-exposure fading; simple and easy to be used in numbers on and in organ depths so that preferably all the dosimeters can be exposed under one single exposure to avoid busy beam time.

Neutron dosimetry in mixed radiation fields is rather complex in general and in exotic mixed-field situations when neutron fluence is very low in high-dose low-LET radiation fields such as PNs in high-energy high-dose rate X-ray beams^1–6,8,16^ or deuterium-deuterium fusion neutrons in the presence of low-LET X-rays and electrons as well as high-LET high-fluence ions in plasma focus devices in particular^43^. Regardless of complexities, different interesting methods have been advanced for in and out of the field and/or depth dose studies such as super-heated drop/bubble detectors^4,13–15^; polycarbonate neutron dosimeters (PND)^1–3,16,17^; CR-39/^10^B (with no cadmium cover)^4^; activation of material^2,7,18^; paired ionization chambers^19^; online digital detector in 5 accelerators (15, 18 and 23 MV)^20^; paired ^6^LiF:Mg,Ti and ^7^LiF:Mg,Ti TLDs^21,22^; silicon diodes^23^; BF_3_ and ^3^He proportional counters^24^; and thermal neutron pulse detectors and solid state thermal neutron rate detectors^25^. One extensive study used a digital thermal neutron detector inside a radiotherapy bunker with simultaneous use of passive CR-39 and ^6^Li*/*^7^Li TLD pairs on an anthropomorphic phantom for head and abdomen treatments^20^. In particular, some major studies have been reviewed for in/out-of-field X-rays and neutron dose measurements usually in water tanks for estimating SPC risks by some solid state dosimeters and ion chambers^11^.

Some simulation studies were based on Monte Carlo calculations using codes such as MCNP4 (in soft tissue and water phantoms)^26^ and MCNPX (in a water phantom) for different field sizes^27^, in ICRU tissue phantom in 5 accelerators (15, 18 and 20 MV)^28^ and in a tissue-equivalent phantom^29^.

Neutron spectrometry methods have been rather advanced for in and out-of-field PN spectrometry^1,9,30–33^. However, PN spectrometry in locations requiring high spatial resolution in particular on skin and in tissue-specific organ depths has been a rather challenging requirement. Sohrabi has developed over the last 5 decades some neutron dosimetry/spectrometry methods for different applications such as individual neutron dosimetry, PN dosimetry in medical accelerators, PN spectrometry, PN volume dose equivalent” hypothesis and methodology, mega-size PN dosimetry, PN isodose distributions, etc.^1,2,9,12,16,17,34,35,37–43^. Based on the “lessons learned and experiences gained” on such developments, a rather simple multi-function miniature neutron dosimeter/spectrometer with a high spatial resolution, capable of also detecting albedo neutrons scattered back from body/phantom, was invented and applied in this study which can determine seven major energy-specific PN ambient dose equivalent values at each tissue-specific measurement point on and in phantom organ depths as well as other energy-specific values driven from the major PN dose equivalent values, if it is needed to be used. Therefore, it is the purpose of this paper to:

1. Introduce a “novel miniature neutron dosimeter/spectrometer” (hereafter neutron dosimeter) which uniquely provides seven major energy-specific PN dose equivalent values plus other desired combined values at tissue-specific measurement points for accurate PN-SPC risk estimation or other applications,
2. Determine seven major energy-specific and tissue-specific PN dose equivalent per unit 18 MV X-ray dose responses on and in phantom depths and also in and out-of-field in organs remote from the central axis in human-size polyethylene (PE) phantom including beam thermal, albedo thermal, total thermal, total epithermal, total fast, sum of totals (thermal + epithermal) and sum of totals (thermal + epithermal + fast) PNs dose equivalents for prostate cancer therapy in 10 cm × 10 cm field of 18 MV X-rays linear medical accelerator, and
3. Study, demonstrate, and discuss the unique features of the neutron dosimeter also as a “miniature neutron spectrometer” in obtaining matrix of PN energy-specific and tissue-specific depth dose data and for other applications requiring high spatial resolution in health physics in particular individual dosimetry, medical physics, environment/cosmic-ray physics, space flights, etc.

## Experiments and methods

### Novel miniature neutron dosimeter/spectrometer

#### General characteristics of the dosimeter

This is a simple, efficient and unique miniature neutron dosimeter/spectrometer which can provide neutron energy-specific dose equivalents, as invented and used in this study. It is based on using bare PNDs and PND/^10^B (with one or two cadmium cover), as are discussed in detail below. This neutron dosimeter can determine distinctly seven energy-specific PN dose equivalent values for beam thermal, albedo thermal, total thermal, total epithermal, total fast, sum of totals (thermal + epithermal) and sum of totals (thermal + epithermal + fast) PNs dose equivalents at any measurement point which are of importance in health and medical physics.

The PND studies over decades have shown that the PND has unique neutron dosimetry characteristics. The PND detects efficiently fast-neutron-induced secondary charged particles and performs ideal characteristics such as wide dose range, almost nil fading, high sensitivity to fast-neutron-induced secondary charged particles, broad alpha energy response, nil sensitivity to low-LET radiation (×, β, γ) and non-ionizing radiation, acceptable directional dependence in particular when cylindrical dosimeters are used or when 4π exposure is applied; high spatial resolution, potential to be used from a small to mega size detectors, assessable by the unaided eyes, availability in common plastic markets at very low cost, etc.^17,34,35^. A number of precisely calibrated neutron sources (e.g. fission neutron spectrum; Pu-Be; 16, 35 and 50 MeV d+ on Be targets from 3 different cyclotrons) have been used to determine neutron energy response. In particular, it has been demonstrated that the dosimeter response versus neutron energy match well with ICRP ambient dose equivalent H*(10) (fluence-to-ambient dose equivalent response) from ∼1 to ∼20 MeV; as determined by three independent studies^35^. The conversion factor obtained is constant over the stated energy range making the PND response independent of neutron spectrum, an ideal neutron dosimetry characteristic.

In order to detect also fast neutrons with energy < 1 MeV as well as thermal and epithermal neutrons from the beam and from the phantom sides as albedo neutrons, an albedo neutron dosimeter based on PND/^10^B (with or without cadmium cover) has been invented and advanced over decades for different applications in particular individual dosimetry^17,34–36,39,43,44,50,51^ and PN dosimetry^34,36,39,44^. The PND is also highly sensitive to alpha particles in particular 1.47 MeV thermal/epithermal-neutron-induced alpha particles (with near 100% efficiency) from ^10^B converter generated through ^10^B(n_th_,α)^7^Li reactions with a high cross section of 3840 barn^8,34^. Since a fraction of epithermal PNs is absorbed in cadmium, correction factors of 1.07 and 1.13 were applied respectively when one or two 0.5 mm thick cadmium covers have been used^39,45,52,53^.

The dosimeters have also been further calibrated by a standard ^252^Cf source to further recheck the sensitivity. For thermal and epithermal PN dose equivalent determination, conversion factors were also obtained from extensive fast, epithermal and thermal neutron dosimetry studies of the albedo neutron dosimeters using PND/^10^B (with or without cadmium cover). The dosimeter was calibrated on different phantoms with different calibrated neutron sources such as Pu-Be, Am-Be and in particular ^252^Cf source, even in a scatter free air environment, as well as by simulation yet to be published^36,50,51^.

As regards directional dependency of the dosimeter, the directional dependency is minimized or becomes negligible when the detector size approaches relatively a very small detector compared to large detectors such as spherical detectors. Also the dosimeter receives PNs impinging on it from all directions in 4π space. Especially, each dosimeter placed at a phantom depth is distanced by PE cylindrical separators from 1 to 4 cm. Therefore, each dosimeter receives PNs from the beam and from phantom sides in 4π space from top, bottom and the other sides, which can be considered more or less an isotropic distribution. Such arrangements make the directional dependence of the dosimeter rather low or negligible under such neutron exposure conditions.

As has been stated above, it is generally believed that mixed neutron field dosimetry in particular at extreme conditions like PN dosimetry in high-energy high-dose X-ray beams remains one of the most difficult tasks to accomplish accurately with known uncertainties the degree of which depends on the method applied. The accuracy is usually related to quantitative measure of how values deviate from a standard or expected value. Unfortunately, there is no PN standard dosimetry laboratory and methods as well as any standard dose equivalent value per unit X-ray dose to be used as a main reference for correct estimation of real accuracy. So only very limited investigations on the accuracy of measurements have been performed due to having many parameters involved in mixed field dosimetry. Researchers using simulation methods believe that the most accurate techniques cannot achieve an uncertainty better than 10%^29^. Some researchers also believe that the most elaborate techniques rarely permit an accuracy better than 10%^54^. Some studies by considering some parameters roughly estimated uncertainties up to 20%^4,54,55^.

Considering our neutron dosimetry method with total insensitivity to high-dose-high energy X-rays, long neutron energy ICRP ambient dose equivalent response, and optimized electrochemical etching (ECE) methods with well experienced operators, the method experiences some uncertainties in parameters such as neutron energy response conversion factors (< 5%), X-ray dose delivered (< 2%), ECE processing (< 4%) with dose equivalent determination within one standard deviation (σ) lead to an overall estimated accuracy of < 20%. Such exercises on accuracy determination need to be further advanced in PN dosimetry.

### Neutron dosimeter/spectrometer design and performance

**Figure 1:**
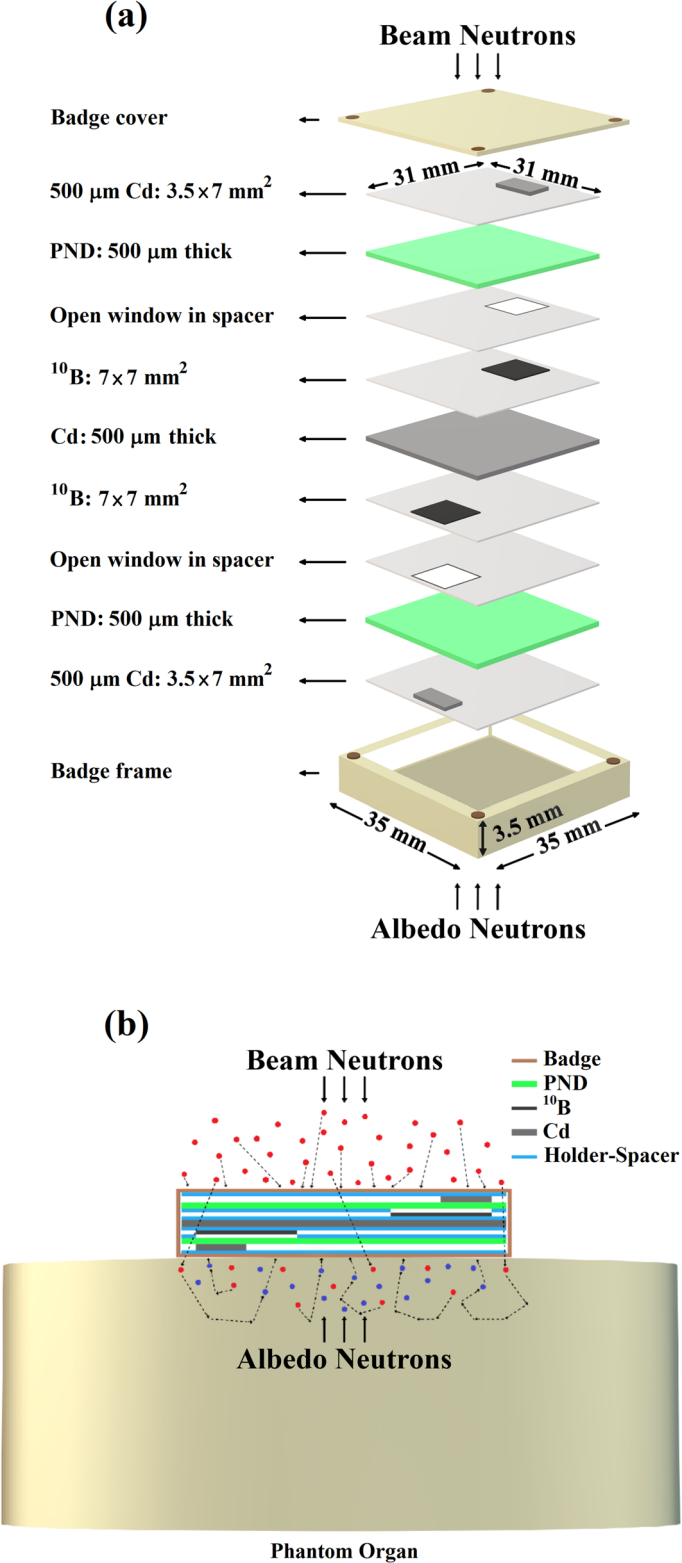
(**a**) Schematic components of the “miniature neutron dosimeter/spectrometer”, and (**b**) the dosimeter components assembled in a badge as placed on a phantom.

Figure 1a,b shows the schematic components of the “miniature neutron dosimeter/spectrometer” (a), and the dosimeter components assembled in a thin Plexiglas badge and placed on a phantom for full performance (b). The dosimeter basically consists of ECE-processed PND, with a chemical formula (C_16_H_14_O_3_)n almost resembling the skin, for fast neutron dosimetry and PND/^10^B (with one or two cadmium cover) for thermal and epithermal PNs from the beam and from scattered albedo PNs, as described below^17,34,35^.

1. Each of two bare PNDs in the dosimeter basically detects total fast PNs through fast-PN-induced secondary charged particle tracks (called recoils) from beam and from albedo phantom sides based on which total recoil track density is determined. Therefore, the bare PND detects total fast PNs from both beam and phantom sides.
2. Each PND also faces a ^10^B converter chip (0.7 cm × 0.7 cm size) fixed on a plastic holder making overall two independent PND/^10^B dosimeters on either side of the middle cadmium foil. Therefore, the PND under ^10^B converter detects thermal/epithermal-PN-induced alpha particles in addition to fast-neutron-induced recoil tracks. A PND/^10^B with no cadmium cover can detect thermal, epithermal and fast PNs, However, when having cadmium cover inserted with different arrangements, the response will be different.
3. The PND on either side is separated from the ^10^B converter by 125 µm thick plastic spacer with an opening of 0.7 cm × 0.7 cm to allow thermal/epithermal-induced-alpha particles to pass and imping the PND. This makes an effective PND area of 0.49 cm^2^ on which thermal/epithermal-alpha-induced particle tracks are recorded, in addition to fast-PN-induced recoil tracks. Therefore, the beam PND/^10^B with one cadmium underneath detects beam thermal, total epithermal, and total fast PNs and the PND/^10^B in phantom side having cadmium on top detects albedo thermal, total epithermal and total fast PNs.
4. Each ^10^B converter in either side of the dosimeter also faces an additional external cadmium chip (0.35 cm × 0.7 cm size) fixed on a plastic holder so that it shields half of each ^10^B converter on either side of the middle cadmium from thermal PNs. Accordingly, each PND/^10^B part with cadmium on both sides detects total epithermal and total fast PNs from the beam and phantom sides, but thermal PNs have been shielded out.
5. In order to determine net alpha track density, total recoil track density is deducted from the total recoil and alpha track density recorded under each ^10^B converter on the PND.
6. The beam thermal, albedo thermal, total thermal, total epithermal, total fast, sum of totals (thermal + epithermal) and sum of totals (thermal + epithermal + fast) PN dose equivalents can be determined as seven major PN dose equivalents as follow:
7. Beam thermal PN track density = Net track density of beam PND/^10^B part with one cadmium underneath minus the part with two cadmium covers,
8. Albedo thermal PN track density = Net track density of albedo PND/^10^B part with one cadmium on top minus the part with two cadmium covers,
9. Total thermal PN track density = Beam plus albedo thermal PN alpha track densities,
10. Total epithermal PN track density = Mean of total beam plus albedo alpha track densities PND/^10^B part with two cadmium cover,
11. Total fast PN recoil track density = Mean of recoil track density of two bare PNDs,
12. Sum of totals (thermal + epithermal) = Sum of total thermal plus total epithermal track densities, and
13. Sum of totals (thermal + epithermal + fast) = Sum of total thermal, total epithermal and total fast track densities.

It should be mentioned that the bare PND only detects total fast PNs from the beam side and also from phantom side as one total value. Unlike the PND/^10^B arranged on both sides of the cadmium filter which detects thermal PNs from both sides, the bare PND cannot distinguish fast PNs coming from either side and no mechanism could have been formulated to perform this separation. The same is true with epithermal PNs; PND/^10^B whether used as bare or covered by one or two cadmium covers detects epithermal PNs from the beam and from the phantom side. In fact, the PND/^10^B on both sides of the middle cadmium provide the same epithermal PN results. Therefore, only total epithermal PNs has been detected and reported.

### Dosimeter processing

After the neutron dosimeters have been exposed on surface and in phantom organ depths to PNs, the two PNDs from each dosimeter were removed and processed in the triplet ECE chamber by applying the 50 Hz–2 kV ECE method in an optimized etchant mixture of 45 g water (H_2_O), 40 g ethanol (C_2_H_5_OH), and 15 g potassium hydroxide (KOH) at 26 ± 1 °C for 6 h using a simple 50 Hz—high voltage generator developed in our laboratory^46–49^. It is interesting to note that the ECE-processed secondary charged particle tracks and alpha particle tracks can be easily observed by the unaided eyes so that one can sharply distinguish the areas exposed to fast-PN-induced secondary charged particles and thermal/epithermal-PN-induced alpha particles in different areas of the PND. The mean track densities were determined by counting the tracks under a light microscope. Accordingly, the mean track densities (tracks.cm^−2^) due to fast, epithermal and thermal PNs, after being separately determined as discussed above, are converted to dose equivalent using relevant conversion factors.

### Solid polyethylene human phantom

A solid PE human-size phantom (hereafter called phantom) was designed and built in order to determine PN dose equivalents on and in phantom organ depths as well as in and out-of-field using solid PE blocks with a density of ~1 gr cm^−3^, as close as recommended by ICRP 89^56^. Studies have shown that the PE material has been proved show that the total neutron cross section versus neutron energy for different materials including polyethylene, matches well with that of the standard tissue equivalent material A-150^57^.

**Figure 2:**
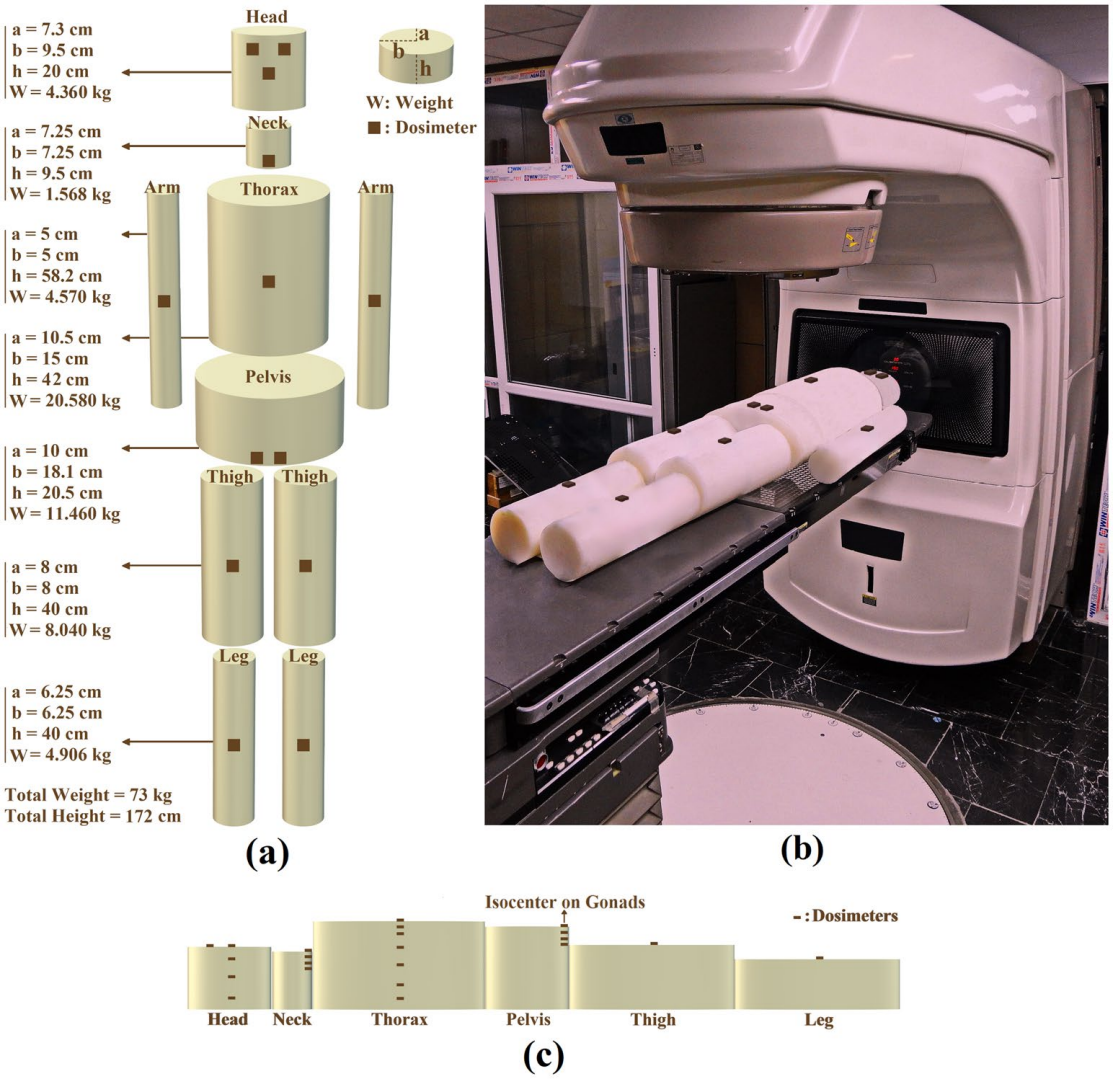
(**a**,**b**,**c**) Solid PE human-size phantom; (**a**) with neuron dosimeters arranged on the surface of organs, (**b**) placed on the patient couch for a 10 cm × 10 cm field of Varian Clinac 2100C medical linear accelerator for prostate cancer treatment with isocenter on gonads, and (c) neutron dosimeters placed on and in phantom organ depths of head, neck (thyroid), thorax and pelvis (gonads).

The phantom consists of ten solid PE cylinders of different shapes which were machine made either as cylinders (neck, arm, thigh and leg) or as elliptical cylinders (head, thorax and pelvis) organs with dimensions and specifications given in Fig. 2a. Figure 2a,b,c shows solid PE human-size phantom; (a) with neutron dosimeters arranged on the surface of the organs, (b) placed on the patient couch for a 10 cm × 10 cm field of Varian Clinac 2100C medical linear accelerator for prostate cancer treatment with isocenter on gonads, and (c) with neutron dosimeters placed on and in phantom organ depths of head, neck (thyroid), thorax and pelvis (gonads).

In order to determine thermal, epithermal and fast PN dose equivalent values on and in phantom organ depths either directed from the beam or scattered back from the phantom layers underneath into the dosimeter as albedo neutrons, 13 neutron dosimeters were placed on the surface of 10 phantom organs; two on eyes, one at the center of head, one on thyroid (neck), one at the center of thorax, one on each arm, two on gonads, one on each thigh and one on each leg, as shown in Fig. 2a.

For determining also thermal, epithermal and fast PN dose equivalent responses on and in depths of four phantom organs head, neck (thyroid), thorax, and pelvis (gonads), hollow cylinders with 4.5 cm diameter were carved through the middle of thorax and head organs as well as at the location of gonads and thyroid on neck and pelvis phantom organs respectively so that the neutron dosimeters can be easily placed at different depths with separated by solid cylindrical PE fillings (Fig. 2c). Four neutron dosimeters were placed at 0, 3.0, 7.3 and 12.6 cm head depths; four dosimeters at 0, 1.5, 3.0, and 4.2 cm in thyroid (neck) depths; seven dosimeters at 0, 1.5, 3.0, 6.5, 10.5, 15.5, and 18.0 cm thorax depths; and four dosimeters in at 0, 1.5, 3.0, and 4.2 cm gonads (pelvis) depths. Every other dosimeter was rotated horizontally by 90° relative to each other to minimize possible shielding effects. Such neutron dosimeter arrangements provide seven PN depth dose equivalent values at each location based on which seven PN depth dose equivalent responses have been determined for each organ.

The phantom with the neutron dosimeters on and in its organ depths was laid on the patient couch of the medical linear accelerator simulating a real patient. The phantom with dosimeters on then was exposed to 10 Gy (1000 MU) of 18 MV X-rays from a Varian Clinac 2100C medical linear accelerator (Fig. 2b). The isocenter was fixed on the gonads with the aim of prostate cancer treatment in a 10 cm × 10 cm field with a source to skin distance (SSD) of 100 cm.

### Experimental findings and results

#### Photoneutron whole body dose equivalent distribution on/in organ depths

**Figure 3:**
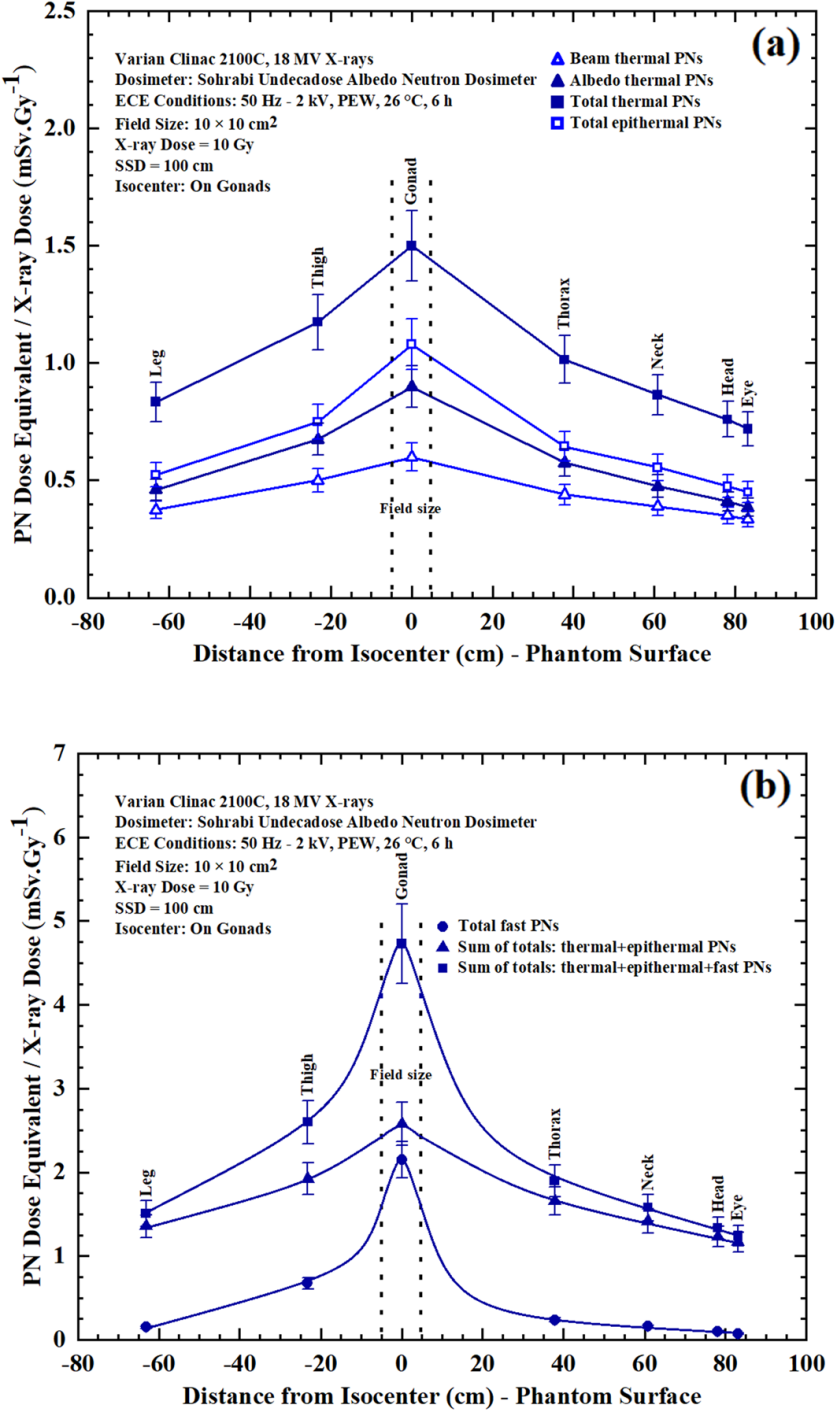
(**a**,**b**). PN dose equivalent/Gy 18 MV X-ray dose (mSv Gy^−1^) versus distance from isocenter (gonads) responses of measurement points on surface of 7 organs for; (**a**) beam thermal, albedo thermal, total thermal, and total epithermal, and (**b**) total fast, sum totals thermal + epithermal and sum total thermal + epithermal + fast PNs for a prostate cancer treatment in 10 cm × 10 cm field of 18 MV X-rays of the medical linear accelerator at a SSD of 100 cm exposed to 10 Gy X-ray dose.

After necessary PN exposures in the beam, ECE processing, and track density assessments, the seven major PN dose equivalent/Gy X-ray dose values were determined at each measurement point. The PN dose equivalent/Gy X-ray dose (mSv Gy^−1^) versus distance from isocenter (gonads) responses of surface measurement points on seven phantom organs are shown in Fig. 3a,b for; (a) beam thermal, albedo thermal, total thermal and total epithermal, and (b) total fast, sum total thermal + epithermal and sum total thermal + epithermal + fast PN dose equivalents. The responses are for the dosimeters placed on the surface of the stated organs for a prostate cancer treatment in 10 cm × 10 cm field of 18 MV X-rays of the medical linear accelerator at a SSD of 100 cm exposed to 10 Gy X-ray dose. It should be mentioned that the data obtained for the eyes, gonads, thighs and legs are the mean values of two similar measurement points at similar locations on the phantom organs.

As seen above in Fig. 3a,b, the seven major PN dose equivalent (mSv)/Gy X-ray doses determined at the gonads’ surface (isocenter) are as given in the brackets for beam thermal (0.60 ± 0.07), albedo thermal (0.90 ± 0.08), total thermal (1.50 ± 0.18), total epithermal (1.09 ± 0.11), total fast (2.15 ± 0.23), sum total thermal + epithermal (2.59 ± 0.29), and sum total thermal + epithermal + fast (4.74 ± 0.49) photoneutrons. In particular, from the seven major energy-specific PN dose equivalents determined, one may also select other combined dose equivalent values in a selected energy range of interest for any specific application.

**Figure 4:**
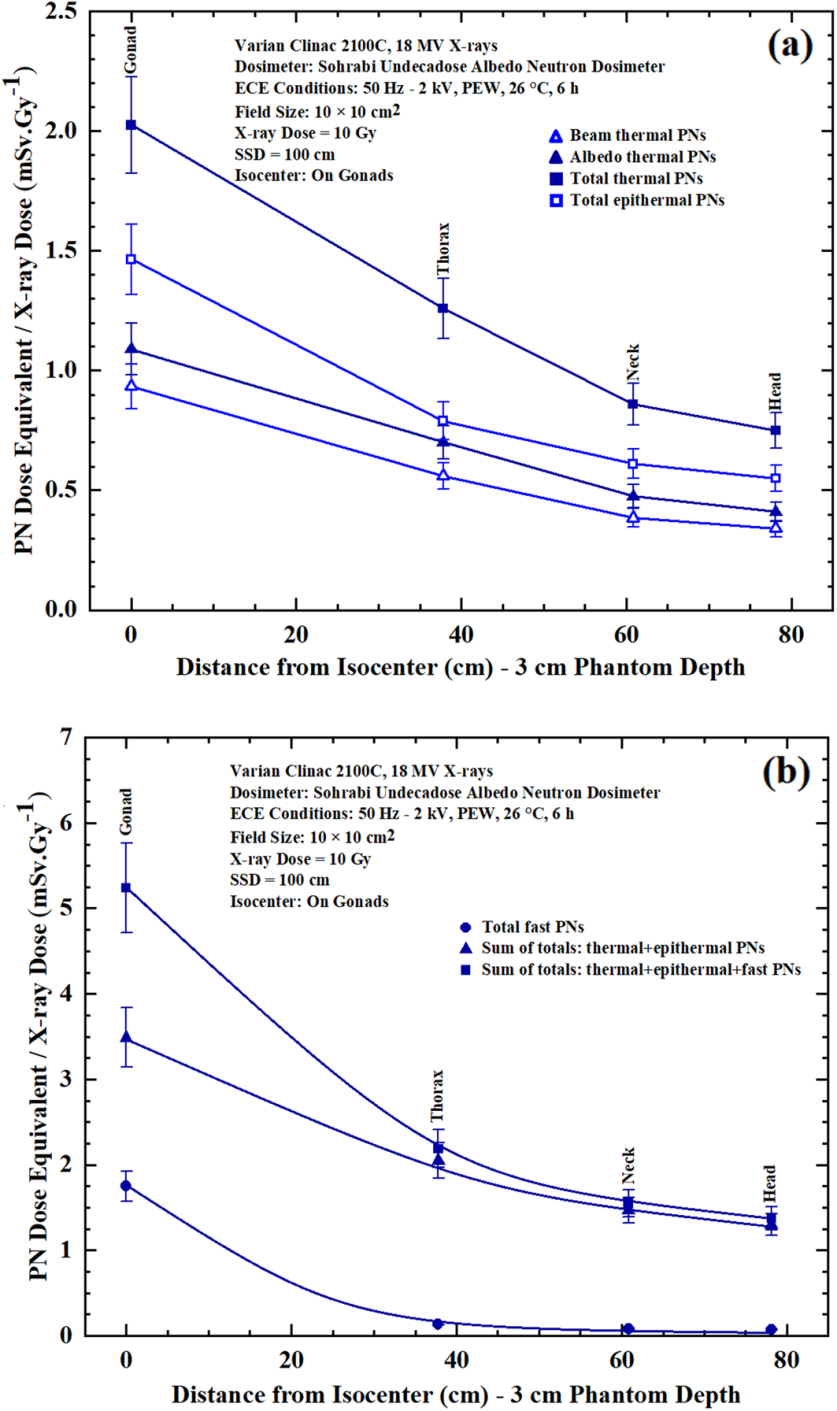
(**a**,**b**) PN dose equivalent/Gy X-ray dose (mSv Gy^−1^) versus distance from the isocenter (gonads) for neutron dosimeters placed at 3 cm depth in gonads, thorax, thyroid and head for a prostate cancer treatment for; (**a**) beam thermal, albedo thermal, total thermal, total epithermal, and (**b**) total fast, sum of totals thermal + epithermal and sum of totals thermal + epithermal + fast PNs in 10 cm × 10 cm field of 18 MV X-rays of the medical linear accelerator at a SSD of 100 cm exposed to 10 Gy X-ray dose.

Photoneutron dose equivalent/Gy X-ray dose (mSv Gy^−1^) versus distance from isocenter (gonads) responses were also obtained at 3 cm depth of some phantom organs including gonads (on pelvis), thorax, thyroid (on neck) and head. Figure 4a,b shows seven PN dose equivalent/Gy X-ray dose (mSv Gy^−1^) versus distance from isocenter responses for neutron dosimeters placed at 3 cm depth in gonads, thorax, thyroid and head for a prostate cancer treatment. It should be noted that thorax, thyroid (neck) and eyes (head) are out of the field and remote from the isocenter (gonads).

By analyzing comparatively the seven PN energy-specific and tissue-specific dose equivalent/Gy X-ray dose responses of Figs. 3a,b and 4a,b, the following conclusions are made:

1. From Fig. 3a,b, it can be seen that fast PN dose equivalent dominates on and in depths under the gonads at the isocenter while it decreases to lower values on the surface and in depths of organs remote from the central axis.
2. The sum total thermal + epithermal + fast, sum total thermal + epithermal and total fast dose equivalent/Gy X-ray dose responses of Fig. 3b have highest values compared to thermal and epithermal responses of Fig. 3a.
3. By comparing the seven PN dose equivalent/Gy X-ray dose responses of Figs. 3a,b and 4a,b , it can be seen that the responses in respect to their values have similar trends in a decreasing order either on the surface or at 3 cm depth.
4. The energy-specific PN dose equivalent/Gy X-ray dose values at each tissue-specific measurement point of responses provide the opportunity to precisely estimate the PN-SPC risk of different tissues in organs of patients undergoing high energy X-ray therapy^11,12,58,59^.
5. It is highly interesting to note that the responses are all in support of what is expected from the beam conditions as regards PNs coming from the beam side and from the phantom side scattered back into the dosimeter.
6. As regards the neutron dosimeter/spectrometer applied, it can be primarily concluded that albeit the neutron dosimeter/spectrometer has a simple design, it is a powerful dosimeter with high neutron sensitivity to distinctly determine seven energy-specific PN dose equivalent/Gy X-ray dose values at each tissue-specific measurement point.
7. It can also be claimed that this simple neutron dosimeter can be considered as a simple “miniature neutron spectrometer” providing detailed energy-specific PN dose equivalent/Gy X-ray dose data compared to the multi-directional spectrometer using 9 polyethylene spheres as we recently invented^9^; i.e. the neutron dosimeter introduced is in fact a multi-function dosimeter with high spatial resolution unique also for PN spectrometry at tissue-specific organ depths.

#### Photoneutron dose equivalent in organ depths

**Figures 5:**
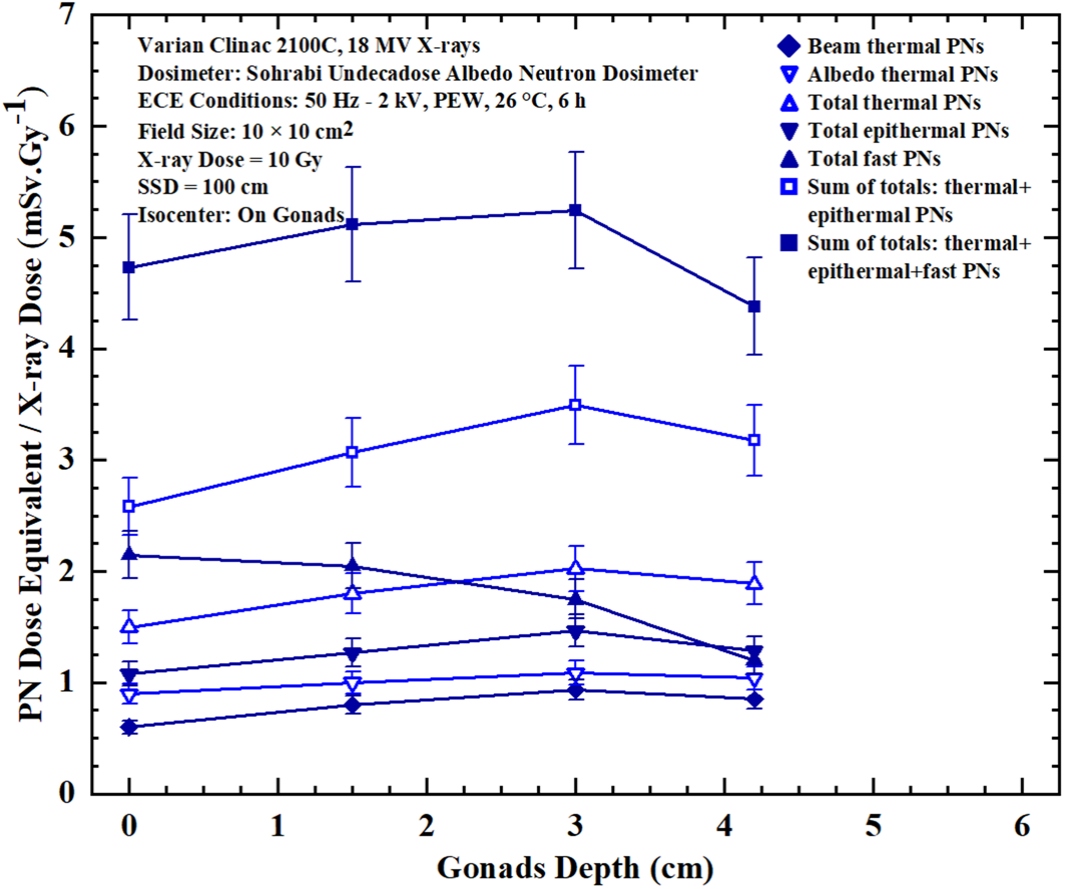
Seven PN dose equivalent/Gy X-ray dose versus depth responses at 0, 1.5, 3.0, 4.2 cm depths under gonads in pelvis phantom organ for seven different dose equivalent components at each measurement depth point for 10 Gy 18 MV X-rays in prostate cancer treatment in a 10 cm × 10 cm field size.

**Figures 6:**
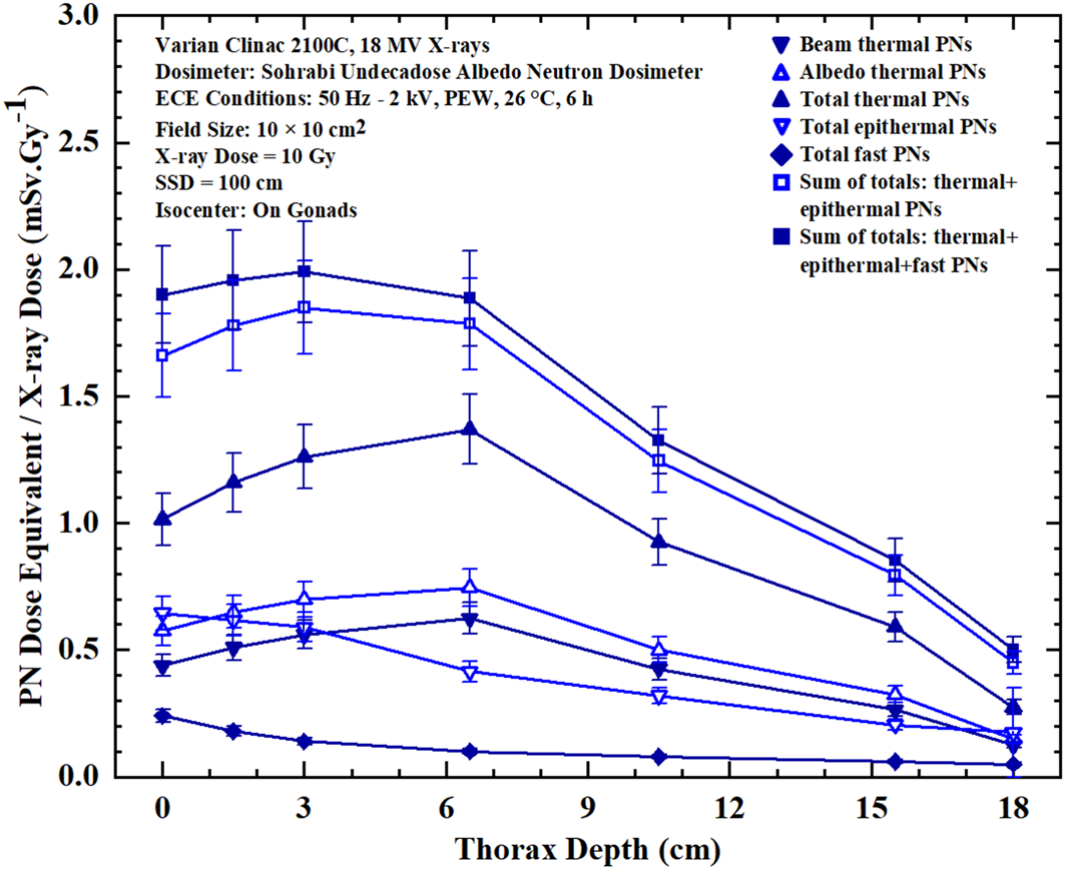
Seven PN dose equivalent/Gy X-ray dose versus depth responses in thorax phantom organ at 0, 1.5, 3.0, 6.5, 10.5, 15.5, 18.0 cm depths at each measurement point for 10 Gy 18 MV X-rays in prostate cancer treatment in a 10 cm × 10 cm field size.

**Figures 7:**
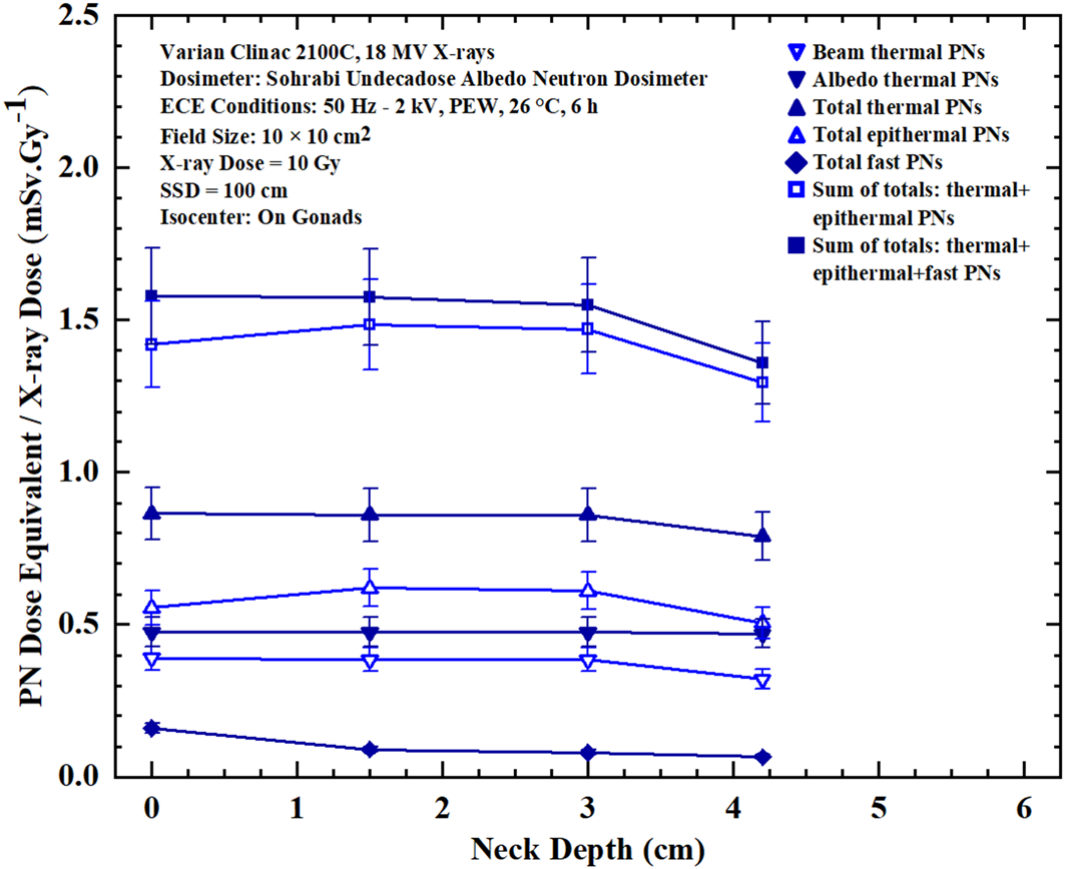
Seven PN dose equivalent/Gy X-ray dose versus depth responses under thyroid in neck phantom organ at 0, 1.5, 3.0, and 4.2 cm depths for seven different dose equivalent components at each measurement point for 10 Gy 18 MV X-rays in prostate cancer treatment in a 10 cm × 10 cm field size.

**Figures 8:**
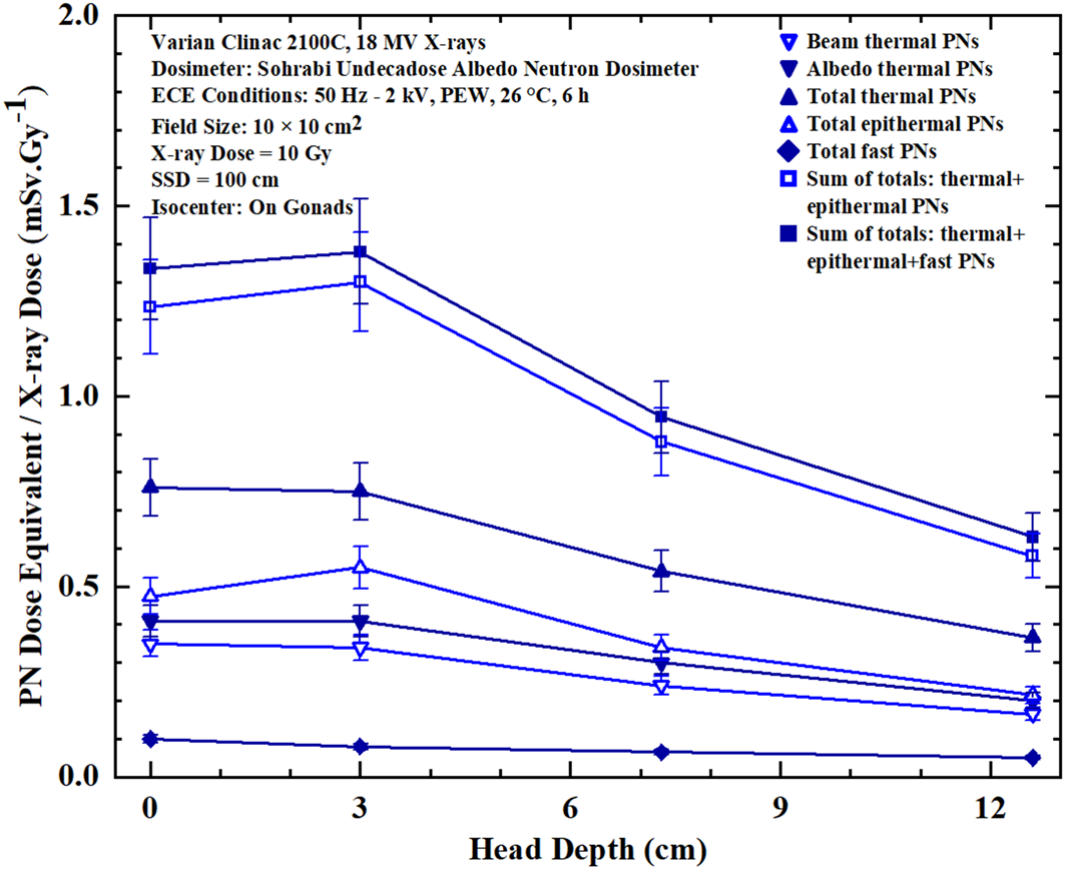
Seven PN dose equivalent/Gy X-ray dose versus depth responses in head phantom organ at 0, 3.0, 7.3 and 12.6 depths for seven different dose equivalent components at each measurement point for 10 Gy 18 MV X-rays in prostate cancer treatment in a 10 cm × 10 cm field size.

When tissue or organ of a patient, which is in this case the prostate, are exposed in a 10 cm × 10 cm field of 18 MV X-rays of the medical linear accelerator at a SSD of 100 cm, tissues in other organs of the patient will also be exposed to PNs. In this context, tissue-specific PN dose equivalent on and in organ tissue depths is of prime importance to be determined in order to estimate the PN-SPC risks of PNs of specific energies. The neutron dosimeters applied provided the opportunity to determine PN dose equivalents/Gy X-ray dose at any desired tissue-specific point on and in depths of other phantom organs out of the field and even well away from the main field. Accordingly, seven PN dose equivalent/Gy X-ray dose responses on and in depths of phantom organs including gonads (pelvis), thorax, thyroid (neck) and head, neutron dosimeters were placed at different depths of the stated phantom organs. For this purpose, cylindrical holes have been carved at the center of four phantom organs such that the dosimeters could be embedded at different tissue depths with spacers. Then, seven PN dose equivalent/Gy X-ray dose responses for each dosimeter embedded at a certain depth in the organ were determined. Figures 5,6,7 and 8 show seven PN dose equivalent/Gy X-ray dose versus depth responses in phantom organs pelvis, thorax, neck and head respectively for prostate cancer treatment in a 10 cm × 10 cm field size, as detailed above.

By analyzing the seven PN dose equivalent (mSv)/Gy X-ray dose responses of Figures 5,6,7 and 8 comparatively, some conclusions follow:

1. The neutron dosimeters have effectively determined beam thermal and albedo thermal PNs separately, total thermal, total epithermal and total fast PN dose equivalent/Gy X-ray dose values from the beam side and phantom side into the dosimeter as well as sum of the relevant values leading to seven major dose equivalent values at each tissue-specific point based on which seven PN dose equivalent/Gy X-ray dose responses on and in phantom depths were determined.
2. The total fast PN depth dose equivalent/Gy X-ray dose responses of Figs. 5,6,7 and 8 in the four organs studied behave differently between themselves and also when compared with other PN dose equivalent responses of other organs. For example, for gonads at the isocenter, fast PN dose equivalent is maximum on the surface and decreases rapidly as depth increases; a trend different from other thermal and epithermal PN responses (Fig. 5). On the other hand, thermal and epithermal PN dose equivalents/Gy X-ray dose prevail in other 3 organs since fast PNs are thermalized by interactions before reaching the thorax, thyroid and head. Therefore, fast PN dose equivalent/Gy X-ray dose responses in thorax, neck and head organs have the lowest dose equivalent/Gy X-ray dose values at the surface and in particular in depths, as expected.
3. This simple and unique neutron dosimeter has been effectively used on the surface and at depths of phantom organs and simply determined seven energy-specific PNs dose-equivalents/Gy X-ray dose directed to the dosimeters from the beam side and from the phantom side scattered back into the dosimeter.
4. As also commented above, the seven major energy-specific PN dose equivalent/Gy X-ray dose values determined at each tissue-specific point provide the opportunity to precisely estimate the energy-specific and tissue-specific PN-SPC risks in organs of patients undergoing high energy X-rays therapy.

## Discussions

Accurate determination of energy-specific and tissue-specific PN dose equivalents in particular for estimating tissue-specific PN-PSC risks are of prime importance requiring dosimeters with high spatial resolutions and capable of providing PN energy-specific dose equivalents^11,12^. On the other hand, while neutron spectrometry has been rather advanced^1,9,30–33^, for obtaining energy-specific dose equivalent data for tissue-specific depth dose studies, neutron spectrometers with high spatial resolution is also required. The neutron dosimeter and methods applied here show how PNs interact and behave after being emitted from the beam and when interact in the patient’s body.

As stated in the introduction, in spite of important scientific and technical advances made on PN dosimetry^1–4,13–15,18–22,24,25,33^, having a neutron dosimeter/spectrometer with high spatial resolution to provide also energy-specific and tissue-specific dose equivalents of PNs generated from the beam and from the phantom or body has been of vital need. In this context, the neutron dosimeter/spectrometer and methods introduced in this study while well meeting such requirements have been extremely instrumental in providing matrix of energy-specific and tissue specific depth PN dose equivalent data, as follow:

1. Detailed matrix of whole body energy-specific and tissue-specific PN dose equivalent/Gy X-ray dose data responses on and in phantom organ depths as well as in/out-of-field remote organs away from the central axis were determined which can be applied for energy-specific and tissue-specific PN-SPC risk estimation.
2. Extensive data obtained demonstrated that gonads being directly at the isocenter of a 10 cm × 10 cm 18 MV X-ray beam receive maximum PN dose equivalents in all seven PN responses of Fig. 3a,b. On the other hand, the out-of-field organs at remote distances from the central axis such as eyes on one side and legs on the other side receive minimum PN dose equivalents.
3. The data obtained demonstrated the potential to specify which types of PNs and within what energy range are directed from the beam, from tissue layers underneath the dosimeter as albedo PNs, and sum total values; what are unique and of importance in medical physics, health physics and other similar applications.
4. This simple neutron dosimeter by providing detailed matrix of energy-specific PN data, has also served as a simple “miniature neutron spectrometer” with high spatial resolution for provision of detailed energy-specific PN dose equivalent data on and in organ depths, which is a rather difficult task.
5. Dose equivalents of PNs from 100 keV to 1 MeV, which is planned in our current studies, by can also be determined by adding a 500 µm thick CR-39 (neutron energy detection threshold > 100 keV) in the dosimeter design.
6. The small 4 cm × 4 cm size dosimeter with only 3.5 cm thickness provides a high spatial resolution neutron dosimetry method for energy-specific and tissue-specific depth dose equivalent studies. In fact, in some pioneering studies, even 6 µm polycarbonate detectors have been applied for neutron air-tissue and bone-tissue interface dose studies^16^. Such spatial conditions may not be obtained by some methods such as PE spheres with thermal detectors at the center namely activation foils, TLDs, ^3^He/BF_3_ proportional counters, and other bulky detectors.
7. Little/negligible post-exposure fading is of prime requirement for individual monitoring services, intercomparison studies of PNs in different medical linear accelerators, etc., and makes the dosimeter also of high commercial value for provision of services.
8. Aside from the initial cost of a simple 50 Hz-HV generator, a Plexiglas ECE chamber, and a microscope, the dosimeter/spectrometer is rather simple with low cost having only one cadmium foil and two small cadmium chips, two small ^10^B converter and two small size 3.1 cm × 3.1 cm PNDs with 500 µm or any other optimum thicknesses. After exposure and processing, only the PNDs should be replaced with almost nil cost. An automatic track counting system may or may not be used in particular for small applications for which track counting under a microscope will be sufficient. A 120 cm × 180 cm 500 µm thick polycarbonate sheet with a high quality masked on both sides, making 2400 PNDs of 3.1 cm × 3.1 cm size, is commercially available in polymer markets for about 10 USD. This cost is almost nil for mass production of dosimeters; only one time cost of one cadmium foil and two ^10^B converter can be considered as initial dosimeter cost for each dosimeter. Such simple requirements mostly home-made at low cost may be compared with high costs of TLD reading system, advanced γ spectrometry system for activation detectors, etc.
9. The dosimeter is in fact quite simple for mass production in any developed and developing laboratories for wide-scale applications in particular for individual neutron dosimetry. Only simple home-made equipment is required for neutron dosimetry, and some other applications such as radon monitoring, individual monitoring, workplace monitoring, etc. since polycarbonate is also efficient for ion detection such as protons, alphas, heavier ions, etc.
10. The seven PN dose equivalents determined are in particular of prime importance for individual neutron dosimetry. The individual dosimeters used by radiation workers at the Oak Ridge National Laboratory in USA had inside it many dosimeter components such as threshold activation detectors in order to estimate neutron spectrum in a reactor or criticality incidents each of which required very sophisticated counting systems. All such needed data can be simply determined by this humble simple miniature neutron dosimeter by using a simple light microscope. Such characteristics make the dosimeter unique for many applications in particular for individual neutron dosimetry a simple version of which was originally proposed and applied for monitoring services^34–36,44,50,51^.
11. More conclusions have been given in the text above and below, on the analysis of the seven PN dose equivalent responses on whole body surface (Fig. 3a,b), at 3 cm depth (Fig. 4a,b) and on and in organ depths remote from the central axis Figs. 5,6,7 and 8.

## Conclusions

A “novel miniature neutron dosimeter/spectrometer” was invented and applied for the first time to PN whole body energy-specific and tissue-specific depth dose equivalent studies simulated by a PE phantom for prostate cancer treatment in 10 cm × 10 cm field of 18 MV X-ray exposure in a medical linear accelerator. This neutron dosimeter/spectrometer and the methodologies introduced are powerful state-of-the-art advances with a simple design consisting of a cadmium foil sandwiched between two PCTD/^10^B dosimeters with two external cadmium chip inserts. This neutron dosimeter proved allowing simply determination of seven major energy-specific PN dose equivalent (mSv)/Gy X-ray dose values at each tissue-specific measurement point for beam thermal, albedo thermal, total thermal, total epithermal, total fast, sum total thermal + epithermal and sum total thermal + epithermal + fast PNs. Accordingly, seven energy-specific PN dose equivalent (mSv)/Gy X-ray dose responses versus distance remote from gonads on the whole body surface length and at 3 cm depth as well as seven tissue-specific depth dose responses in 4 organs in and out of the field remote from the central axis were determined. The matrix of energy-specific and tissue-specific PN dose equivalent (mSv)/Gy X-ray dose responses provides high importance for estimating tissue-specific PN-SPC risks of tissues being directly in and out of the field even in remote locations from the central axis on the surface and in organ depths. In particular, the responses shown in the figures above illustrate highly interesting and valuable data on how PNs are degraded in terms of fluence and energy in particular in remote points from the isocenter on and in tissue-specific organ depths.

The matrix of energy-specific PN dose equivalent (mSv)/Gy X-ray dose responses reported are unique and indicate, among many other specific characteristics stated above, that the neutron dosimeter also simultaneously serves as a simple “miniature neutron spectrometer”. The novel dosimeter/spectrometer introduced is simple, efficient and unique for PN-SPC risk estimation and many other applications in health physics and medical physics in particular individual dosimetry, environment/cosmic-ray physics, space flights, nuclear engineering as well as in science and technology in any developed and developing laboratory.
